# ASO Author Reflections: Composite Endpoint of Liver Surgery: Redefining Metrics for Clinical Trials

**DOI:** 10.1245/s10434-025-17049-7

**Published:** 2025-02-17

**Authors:** Jun Kawashima, Timothy M. Pawlik

**Affiliations:** 1 Department of Surgery, The Ohio State University Wexner Medical Center and James Comprehensive Cancer Center, Columbus, OH USA; 2 Department of Gastroenterological Surgery, Yokohama City University School of Medicine, Yokohama, Japan

## Past

Liver resection remains a cornerstone treatment for primary and secondary liver malignancies.^1^ However, perioperative morbidity represents an ongoing challenge, adversely impacting patient quality of life, prolonging hospital stay, increasing readmission, and compromising long-term survival.^2^ Despite advancements in surgical and perioperative care, the complexity of liver surgery necessitates reliable methods for assessing surgical quality.^3^ Conventional individual endpoints, such as blood loss, specific morbidity and mortality rates, reoperation, and hospital length of stay (LOS), while informative, often fail to capture the multifaceted nature of surgical outcomes, particularly as these discrete events occur infrequently, necessitating prohibitively large sample sizes for robust clinical trials.^3,4^ Recognizing these challenges, our study aimed to develop and validate the Composite Endpoint of Liver Surgery (CELS), an innovative framework to consolidate critical perioperative complications, thereby enabling a more comprehensive assessment and improving the feasibility of high-level evidence generation in liver surgery.^5^

## Present

In the current study, CELS was defined based on the occurrence of one or more critical complications, including blood loss ≥ 2000 mL, bile leak, post-hepatectomy liver failure (PHLF), and post-hepatectomy hemorrhage (PHH). These criteria were identified through univariable analysis for their association with surgery-related death and prolonged hospital stays (LOS). CELS demonstrated robust discriminative ability with an area under the curve (AUC) value of 0.79 in the analytic cohort and 0.85 in the external validation cohort to predict surgery-related mortality. Furthermore, patients with a CELS-positive status experienced prolonged LOS compared with CELS-negative patients (analytic cohort: 14 vs. 9 days, *p* < 0.001; validation cohort: 10 vs. 6 days, *p* < 0.001). Importantly, CELS reduced sample size requirements for clinical trial planning by 45.8–91.6% compared with using individual endpoints alone. This marked reduction in sample size underscores the clinical and scientific utility of CELS, positioning it as an important tool to evaluate liver surgery outcomes.

## Future

CELS offers several distinct advantages over traditional individual endpoints, presenting a transformative opportunity for advancing liver surgery research. By integrating key liver surgery-specific complications, CELS enables a comprehensive evaluation of perioperative outcomes while maintaining a strong clinical relevance through its association with surgery-related mortality. Additionally, its ability to enhance statistical power and significantly reduce sample size requirements renders it a practical and efficient endpoint for clinical trials. Incorporating CELS into prospective studies has the potential to accelerate the development of innovative surgical techniques and strategies, particularly in an era characterized by rapid medical advancements. By providing a robust, clinically meaningful, and universally applicable measure of surgical quality, CELS could serve as a critical framework to guide future research and ultimately improve patient outcomes in liver surgery.

## References

[1] Endo Y, Tsilimigras DI, Woldesenbet S, et al. The complication-overall survival (CompOS) risk tool predicts risk of a severe postoperative complications relative to long-term survival among patients with primary liver cancer. *J Gastrointest Surg*. 2024;28(2):132–40. 10.1016/j.gassur.2023.12.010. 38445934 10.1016/j.gassur.2023.12.010

[2] Endo Y, Tsilimigras DI, Munir MM, et al. Machine learning models including preoperative and postoperative albumin-bilirubin score: short-term outcomes among patients with hepatocellular carcinoma. *HPB (Oxford)*. 2024;26(11):1369–78. 10.1016/j.hpb.2024.07.415. 39098450 10.1016/j.hpb.2024.07.415

[3] van den Broek MA, van Dam RM, van Breukelen GJ, et al. Development of a composite endpoint for randomized controlled trials in liver surgery. *Br J Surg*. 2011;98(8):1138–45. 10.1002/bjs.7503. 21557208 10.1002/bjs.7503

[4] Nickel F, Kuemmerli C, Müller PC, et al. The pancreatic surgery composite endpoint PACE—development and validation of a clinically relevant endpoint requiring lower sample sizes. *Ann Surg*. 2024;281(3):496–500. 10.1097/SLA.0000000000006194. 38214195 10.1097/SLA.0000000000006194 PMC11809732

[5] Kawashima J, Akabane M, Endo Y, et al. The composite endpoint of liver surgery (CELS): development and validation of a clinically relevant endpoint requiring a smaller sample size. *Ann Surg Oncol*. 2025. 10.1245/s10434-025-16965-y. 39888467 10.1245/s10434-025-16965-y PMC11976826

